# Evolution of organismal stoichiometry in a long-term experiment with *Escherichia coli*

**DOI:** 10.1098/rsos.170497

**Published:** 2017-07-19

**Authors:** Caroline B. Turner, Brian D. Wade, Justin R. Meyer, Brooke A. Sommerfeld, Richard E. Lenski

**Affiliations:** 1 Ecology, Evolutionary Biology and Behavior Program, Michigan State University, East Lansing, MI, USA; 2 BEACON Center for the Study of Evolution in Action, Michigan State University, East Lansing, MI, USA; 3 Department of Plant, Soil and Microbial Sciences, Michigan State University, East Lansing, MI, USA; 4 Department of Integrative Biology, Michigan State University, East Lansing, MI, USA; 5 Department of Microbiology and Molecular Genetics, Michigan State University, East Lansing, MI, USA; 6 Department of Microbiology and Molecular Genetics, University of Pittsburgh, Pittsburgh, PA, USA; 7 Division of Biological Sciences, University of California, San Diego, La Jolla, CA, USA

**Keywords:** carbon limitation, *Escherichia coli*, experimental evolution, stoichiometry

## Abstract

Organismal stoichiometry refers to the relative proportion of chemical elements in the biomass of organisms, and it can have important effects on ecological interactions from population to ecosystem scales. Although stoichiometry has been studied extensively from an ecological perspective, much less is known about the rates and directions of evolutionary changes in elemental composition. We measured carbon, nitrogen and phosphorus content of 12 *Escherichia coli* populations that evolved under controlled carbon-limited, serial-transfer conditions for 50 000 generations. The bacteria evolved higher relative nitrogen and phosphorus content, consistent with selection for increased use of the more abundant elements. Total carbon assimilated also increased, indicating more efficient use of the limiting element. We also measured stoichiometry in one population repeatedly through time. Stoichiometry changed more rapidly in early generations than later on, similar to the trajectory seen for competitive fitness. Altogether, our study shows that stoichiometry evolved over long time periods, and that it did so in a predictable direction, given the carbon-limited environment.

## 1. Introduction

At the coarsest level, organisms consist of a mixture of elements in varying proportions. While all life shares broadly similar elemental profiles, the ratios vary substantially both inter- and intra-specifically [1–5]. Over several decades, the field of ecological stoichiometry has established that stoichiometric variation has important ecological consequences. However, much less is known about the evolutionary origins of variation in organismal stoichiometry. Addressing this issue is important because evolved changes in organismal stoichiometry might affect ecosystem processes [6] and shape responses to anthropogenic changes [7]. Previous experiments have demonstrated evolved changes in organismal stoichiometry in response to selection on traits such as fecundity [8], temperature adaptation [9] and lipid content [10]. Here, we focus on the evolution of stoichiometry in response to elemental availability itself.

The relative availability of elements in the environment plays an important role in organismal stoichiometry. When a particular element is scarce, the proportion of that element in biomass generally declines, although the degree of physiological plasticity varies among organisms and across elements [5]. However, the effect of elemental scarcity on the evolution of organismal stoichiometry is not well established. All else being equal, one might expect selection for efficient resource use, or ‘elemental sparing’, such that the proportion of a scarce element in biomass would decrease over evolutionary time because organisms that require less of that element can produce more offspring [11,12]. Alternatively, the proportion of a scarce element in biomass might plausibly increase over evolutionary time if that element is necessary for performing an important function in the selective environment. In that case, the benefit of increasing the proportion of that element could outweigh selection for elemental sparing [11]. The proportion of a scarce element might also increase if the evolution of improved mechanisms to acquire that element alleviates its scarcity, leading to a higher proportion of the element in the biomass of evolved organisms compared with their ancestors [13].

Current evidence in support of the evolution of elemental sparing is mixed. The stoichiometry of phytoplankton appears to reflect oceanic stoichiometry over geological time scales [14,15]. Several comparative studies of the elemental composition of proteins and other cellular components also support the elemental-sparing hypothesis. For example, terrestrial plants, whose growth is often limited by nitrogen, show evidence of nitrogen sparing in their genomes and proteomes [16,17] (but see [18]). In both *Escherichia coli* and yeast, proteins involved in the acquisition of nitrogen, phosphorus and sulfur each have a lower content of the corresponding element than do other proteins in those organisms [19]. This pattern suggests that selection acted to reduce the use of a particular element in proteins expressed when that element is scarce. Other examples of microbial elemental sparing are reviewed by Merchant & Helmann [12].

However, there is no evidence of elemental sparing in the few studies that directly compare either the stoichiometry of evolved and ancestral organisms or the stoichiometry of organisms experimentally evolved under different levels of elemental availability. No significant changes in stoichiometry were observed in experiments with bacteria [20], yeast [21], *Daphnia* [22,23] or rotifers [24]*.* Furthermore, two experiments showed the opposite evolutionary response to that predicted by elemental sparing*. Ostreococcus* phytoplankton cultured with high CO_2_ concentrations evolved a lower C : N ratio than *Ostreococcus* cultured with low CO_2_ levels [25]. Similarly, Bragg & Wagner [13] found that proteins whose expression decreased in yeast evolved under carbon limitation were disproportionately carbon poor compared with the rest of the proteome. They suggested that the evolution of improved uptake of carbon might have alleviated the degree of carbon limitation, thereby allowing reduced reliance on carbon-poor proteins.

The contrasting results between the comparative (e.g. [16,19]) and experimental (e.g. [20,21]) studies might be a consequence of differences in time scales. While the comparative studies reflect at least many thousands of years of evolutionary history, the experimental studies cited above lasted only hundreds of generations. More time might be needed for elemental sparing to evolve. Longer time scales may also be more relevant for the evolution of stoichiometry in natural systems. Species sorting and phenotypic plasticity could dominate short-term responses, while evolutionary responses typically require longer periods of time. Indeed, stoichiometry varies widely among bacterial species [26] and bacteria also exhibit plasticity in their stoichiometry in response to varying nutrient availability [27].

To examine the potential for long-term evolutionary changes in organismal stoichiometry, we compared the carbon, nitrogen and phosphorus content of biomass in ancestral and evolved *E. coli* cells from a 50 000-generation laboratory evolution experiment [28–30]. We predicted that the evolved bacteria would have lower carbon content, relative to nitrogen and phosphorus, compared to the ancestral strain for two reasons. First, under the evolutionary conditions, population size is limited by carbon availability and the medium contains high concentrations of nitrogen and phosphorus [29]. All else being equal, we expected that such conditions would select for reduced use of carbon in biomass, as predicted by the elemental-sparing hypothesis. Similarly, the high concentrations of nitrogen and phosphorus should release the bacteria from any prior selection for reduced use of those elements. Second, the bacteria undergo daily batch transfer to fresh medium, a condition that selects for faster growth [31]. Extending the logic of the growth-rate hypothesis [5], we expected that higher phosphorus content at stationary phase would be favoured because it would enable the bacteria to resume rapid growth after the daily transfers into fresh medium.

## 2. Material and methods

### 2.1. Long-term evolution experiment

The long-term evolution experiment (LTEE) was founded in 1988 with *E. coli* B strain REL606 [29,32]. Six of the 12 populations started directly from REL606; the other six began with a mutant clone, REL607, that differed by a neutral marker. The populations have been maintained in Davis–Mingioli minimal medium [33] supplemented with 25 mg glucose and 2 mg thiamine per litre (DM25). The bacteria can use both glucose and thiamine as carbon sources, but not citrate (another component of DM25, which aids iron acquisition, that the ancestral strain and all but one of the evolved populations cannot use). Given the glucose and thiamine in DM25, the molar ratios of C : N : P available to the bacteria are 1 : 17 : 50. For the one population, designated Ara-3, that evolved the ability to consume citrate [34,35], the available C : N : P ratios are 11 : 10 : 30. Each population is serially propagated by a 1 : 100 dilution into fresh medium each day; it then grows 100-fold until the available carbon is depleted, and it thus undergoes approximately 6.6 generations (i.e. doublings) per day. Samples of each population are frozen in 13% glycerol at −80°C every 500 generations, where the cells remain viable. Portions of these frozen cultures can be revived, allowing direct comparison of ancestral and evolved bacteria. Lenski *et al.* [29] provide more details about the methods used in the LTEE.

### 2.2. Sample collection for stoichiometric analyses

We used a paired sampling design in which a clone isolated from each of the 12 populations at 50 000 generations was paired with its ancestral clone, either REL606 or REL607. Each clone was revived from a frozen stock and conditioned for 1 day in Luria–Bertani medium [36]. After an additional 24 h of conditioning in DM25, each clone was transferred, via a 1 : 100 dilution, to 500 ml of DM25. Samples for stoichiometric analyses were collected during stationary phase after 24 h, consistent with the timing of daily transfers in the LTEE. This sampling time had two advantages. First, the DM25 medium supports a low population density (by microbiological standards, approx. 5 × 10^7^ cells ml^−1^); by sampling populations at or near their maximum density, we obtained more biomass and therefore better estimates of their elemental composition. Second, different clones reach mid-exponential phase at different times, depending on their growth rates, which would complicate efforts to standardize procedures.

Each culture was split into two 250 ml aliquots, which were filtered through pre-combusted glass-fibre filters with 0.7 µm pore size. In the case of population Ara-3, which evolved the ability to consume citrate, we filtered only 125 ml due to its increased population density. Each filter was rinsed with an additional 250 ml of 0.85% sterile saline solution made with distilled deionized water in order to remove dissolved nutrients present in the medium from the filter. The dry mass of each filter was measured before and after filtering. For each culture, one filter was analysed for phosphorus content and the other for both carbon and nitrogen content.

We performed sample collection on four different dates with six cultures (three pairs of ancestral and evolved clones) filtered on each day. Pairs of ancestral and evolved clones were assigned to sampling dates randomly. On each day, 250 ml of sterile DM25 was filtered through each of two filters and processed identically to all other samples. These filters served as blanks in subsequent analyses.

To confirm that glass-fibre filters effectively collected both evolved and ancestral bacteria, we compared the number of colony-forming units in filtered and unfiltered medium. The filters retained more than 99% of both the ancestral and evolved bacteria.

### 2.3. Elemental analysis

Elemental content was measured by the Nutrient Analytical Services Laboratory at the University of Maryland Center for Environmental Science. Phosphorus mass was determined via extraction to dissolved phosphate followed by molybdenum blue colorimetric analysis [37] using an Aquakem 250 photometric analyzer. Carbon and nitrogen contents were measured by elemental analysis [38] using an Exeter Analytical Model CE-440 Elemental Analyzer.

### 2.4. Nucleic acid content

We measured the nucleic acid content of the ancestral and evolved clones because higher growth rates are associated with higher levels of RNA, which may drive increases in phosphorus content of cells. However, the total nucleic acid content was measured in stationary phase after 24 h of growth in DM25, the same conditions under which we measured elemental content. The bacteria were revived from frozen stocks and conditioned as described above. Cells were then lysed by heating, and their nucleic acid concentration was measured in duplicate using the Quant-iT RiboGreen assay kit.

### 2.5. Temporal trend in stoichiometry

To examine the temporal trend in stoichiometry over the course of the evolution experiment, we also conducted more detailed sampling of one population, designated Ara-1, through time. This population has been the subject of many studies which have focused on a single population from the LTEE [39–43]. We measured the stoichiometry of two clones isolated from this population at each of the following time points: 500, 1000, 1500, 2000, 5000, 10 000, 15 000, 20 000, 25 000, 30 000, 35 000, 40 000, 50 000 and 60 000 generations. (Note: the LTEE had not yet reached 60 000 generations at the time of our across-population analyses.) Each clone was randomly assigned to one of six sampling dates. Sampling was conducted as described above. The 40 000-generation measurements were excluded from our results. Each exhibited a substantially higher biomass than any other clone in the experiment. We subsequently regrew these clones from the same frozen stocks and observed biomass values consistent with the other clones, indicating that our original measurements were caused by contamination or some other error.

### 2.6. Data analyses

All values for biomass, carbon, nitrogen and phosphorus were corrected for background levels by subtracting the corresponding value measured from a blank filter on the same day. Background levels averaged 9.6% of the measured value of P, 0.6% of N and 4.5% of C. Biomass was calculated as the change in dry mass of the filter before and after filtering. The percentages of carbon, nitrogen and phosphorus were calculated as the mass of each element divided by the total biomass. All elemental ratios were calculated as molar ratios.

Two-tailed paired *t*-tests were used to test for differences between evolved and ancestral biomass; nucleic acid content; percentages of C, N and P; and molar C : N, C : P and N : P ratios. The paired *t*-tests were conducted using JMP 9.0 software. To examine whether C : N, C : P and biomass for the 50 000-generation evolved clones were correlated with fitness, we calculated Kendall's *τ*_b_ rank correlation between the average fitness value previously obtained for each of nine LTEE populations at that generation [28] and the elemental ratio and biomass measures for the clone from each population. To test whether there was a trend over time in the Ara-1 population, we calculated the Kendall's *τ*_b_ rank correlation of C : N, C : P and N : P ratios with generation. We calculated rank correlations for both the full dataset and for values from 5000 generations and beyond. We tested the later time points separately to evaluate whether stoichiometry continued to evolve over a longer time frame. We also examined the correlations of the elemental ratios and biomass (the average value for two clones at each time point) with fitness values obtained for Ara-1 at many of the same time points [28] using Pearson's correlation coefficient for these analyses. For the elemental ratio and biomass values at 25 000 and 35 000 generations, we used the fitness data from 24 000 and 34 000 generations, respectively. All correlation analyses were conducted using R 3.1.0 software.

## 3. Results

### 3.1. Stoichiometry across populations

**Figure 1.**
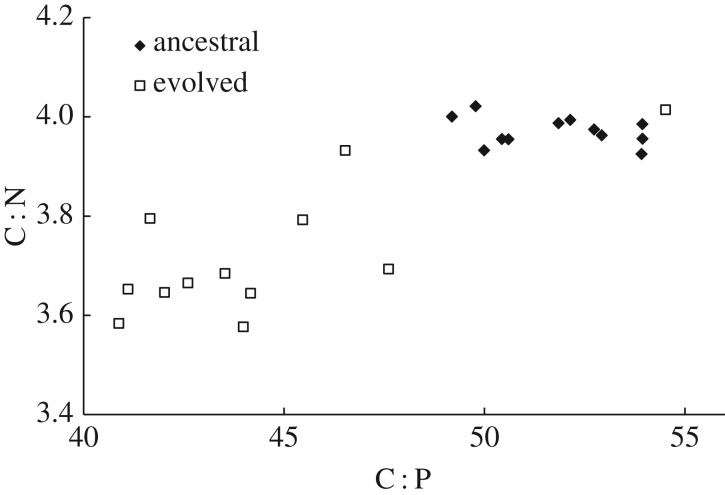
Molar C : N and C : P ratios of the ancestral (filled diamonds) and 50 000-generation evolved (open squares) clones from the LTEE with *E. coli*. The evolved clone at the far upper right is from the only population that evolved the ability to consume citrate; this clone was excluded from the statistical analyses owing to the much higher carbon availability that it experienced.

We observed substantial differences in the ratios of carbon, nitrogen and phosphorus between the ancestral and evolved clones (figure 1). Among the evolved clones, however, one had distinctly different C : N and C : P ratios compared to the other 11, and that one was from the only population that evolved the ability to consume the citrate present in the medium. As explained in the Material and methods section, this population subsequently experienced conditions with much higher carbon availability than the other populations. The sample from this population reached a biomass concentration of 109 mg l^−1^ at 24 h, whereas the samples from the other populations had biomass concentrations between 10 and 18 mg l^−1^. The citrate-consuming clone also had the highest measured C : N and C : P ratios of any of the evolved clones, with ratios as high as or higher than any samples of the ancestral clones. Given the distinctive growth conditions experienced by this clone, we excluded it from our statistical analyses. (Note, however, that inclusion of the citrate-consuming clone does not affect whether the comparisons reported below are statistically significant at the 0.05 level, with two exceptions: the N : P ratio would no longer differ between the ancestral and evolved clones, while their nucleic acid content per biomass would differ.)

**Figure 2.**
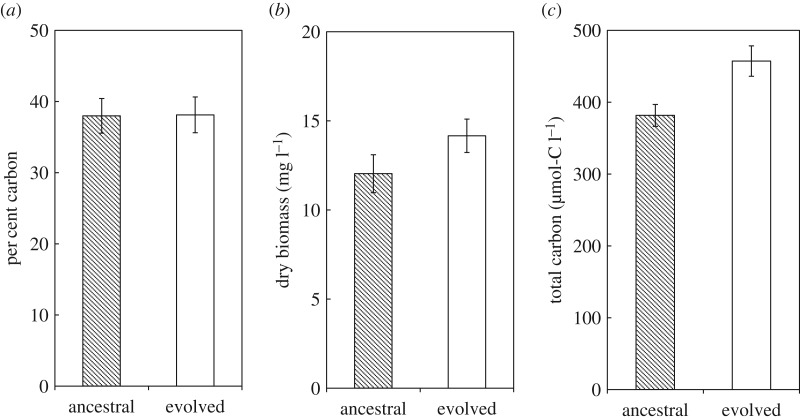
Changes in carbon and biomass. (*a*) The per cent carbon in cellular biomass does not differ significantly between the ancestral and evolved clones (*p* = 0.888). (*b*) Total dry biomass is significantly higher in the evolved clones than in the ancestral clones (*p* < 0.001). (*c*) The total carbon retained in biomass is also significantly higher in the evolved clones (*p* < 0.001). All data shown are means with 95% confidence intervals.

The average molar C : N and C : P ratios both decreased significantly in the evolved clones compared with their ancestors (figure 1, paired two-tailed *t*-tests, C : N *p* < 0.001, C : P *p* < 0.001). Also, the average molar N : P ratio declined from 13.1 to 11.8 (*p* < 0.001). These changes in C : N and C : P ratios were driven by increases in the percentages of both nitrogen and phosphorus in the evolved clones. The percentage of biomass comprised of nitrogen atoms increased from 11.2 to 12.0% (*p* = 0.012), and that of phosphorus increased from 1.9 to 2.3% (*p* < 0.001). The percentage of carbon did not significantly change (figure 2*a*, *p* = 0.888).

The significant increases in the percentages of both nitrogen and phosphorus in the evolved clones' biomass imply that the proportion of one or more other elements must have declined. One possibility is that the carbon content declined slightly, but that the decline was not detected. Supporting this possibility is the realization that an evolved carbon content of 36.7% would be sufficient to offset the combined 1.3% increase in the nitrogen and phosphorus content; that value falls well within the 95% confidence interval (35.6–40.6%) for the average carbon content of the evolved bacteria. Another possibility is a decrease in the proportion of one or more of the unmeasured elements (including hydrogen, oxygen, sulfur and potassium) that together made up approximately 50% of the biomass of the ancestral bacteria.

The C : N and C : P ratios for the population that gained the ability to use citrate are, as noted, quite atypical of the evolved populations. Some of the variation among the other evolved populations is undoubtedly measurement error, as indicated by the variation in these ratios for repeated measurements of the ancestral bacteria (figure 1). Given that the changes in these ratios occurred while the bacteria adapted to the LTEE environment, we asked whether these ratios correlated with competitive fitness for the nine populations for which fitness data were available at 50 000 generations [28]. However, neither the C : N ratio (*τ*_b_ = −0.222, *n* = 9, *p* = 0.404) nor the C : P ratio (*τ*_b_ = −0.056, *n* = 9, *p* = 0.835) showed a significant correlation with fitness.

### 3.2. Biomass and carbon retention

The total bacterial biomass concentration increased by 17.7%, from 12.3 to 14.5 mg l^−1^, in the evolved bacteria (figure 2*b*, *p* < 0.001). Although the percentage of carbon in the biomass did not change significantly, the total amount of retained carbon increased by 17.8%, from 386 to 455 µmol C^−1^ (figure 2*c*, *p* < 0.001), thus mirroring the increase in total biomass. There was no significant correlation between the biomass concentration and competitive fitness at 50 000 generations across the nine populations with available fitness data (*τ*_b_ = 0, *n* = 9, *p* = 1).

### 3.3. Nucleic acid content

The nucleic acid content per culture volume increased 23.6%, from 344 ± 22 µg l^−1^ (mean ± 95% confidence interval) in the ancestors to 425 ± 31 µg l^−1^ in the evolved bacteria (*p* < 0.001). However, most of this change reflected the increase in total biomass. The proportion of nucleic acid in the biomass averaged 28.3 ± 2.5 µg-nucleic acid/mg-biomass in the ancestral clones and 29.6 ± 3.0 µg-nucleic acid/mg-biomass in the evolved clones. This difference was not significant (*p* = 0.116), although the trend was in the predicted direction. Given that nucleic acids are about 8.7% P [5], then the difference in nucleic acid content can account for at most a 0.12 µg-P/mg-biomass increase in phosphorus content, which constitutes only approximately 4% of the observed 3.3 µg-P/mg-biomass increase in phosphorus content. Similarly, the change in nucleic acid content can account for at most 0.2 µg-N/mg-biomass, or approximately 2% of the observed 8.49 µg-N/mg-biomass increase in nitrogen content.

### 3.4. Temporal trajectory of stoichiometry

**Figure 3.**
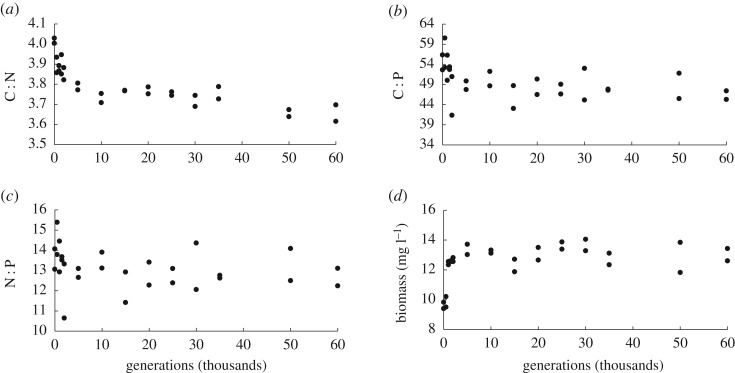
Trajectories for elemental ratios and biomass in population Ara-1 between 0 and 60 000 generations. The (*a*) C : N, (*b*) C : P and (*c*) N : P ratios all declined over time, while the (*d*) dry biomass per volume increased.

The ratios of C : N, C : P and N : P all show the same temporal trends in population Ara-1 as observed across all populations at 50 000 generations (figure 3). In all cases, the change in stoichiometry was faster initially and slower in later generations. Over the entire time course, the C : N (Kendall's *τ*_b_ = −0.760, *n* = 28, *p* < 0.001), C : P (*τ*_b_ = −0.458, *n* = 28, *p* = 0.001) and N : P (*τ*_b_ = −0.291, *n* = 28, *p* = 0.032) ratios all declined significantly with time, while biomass increased significantly (*τ*_b_ = 0.441, *n* = 28, *p* = 0.001). All three elemental ratios and biomass concentration also show significant correlations with fitness values measured over time in population Ara-1 (Pearson's correlation with *n* = 12; C : N *r* = −0.860, *p* < 0.001; C:P *r* = −0.776, *p* = 0.002; N : P *r* = −0.562, *p* = 0.045; biomass *r* = 0.663, *p* = 0.013).

To determine whether the changes in stoichiometry continued after the initial period when stoichiometry changed rapidly, we tested whether the trends were significant from 5000 generations on. The C : N ratio showed a significant decline from generations 5000 to 60 000 (*τ*_b_ = −0.539, *n* = 18, *p* = 0.002). However, C : P (*τ*_b_ = −0.175, *n* = 18, *p* = 0.322) and N : P (*τ*_b_ = −0.067, *n* = 18, *p* = 0.703) did not change significantly over this later period, though both trends were consistent with continued declines. The biomass trend was also not significant over the period from 5000 to 60 000 generations (*τ*_b_ = −0.047, *n* = 18, *p* = 0.789).

## 4. Discussion

Our results demonstrate substantial evolutionary change in the stoichiometry of *E. coli* cells over time (figure 1). The average C : P ratio decreased by 14% and the average C : N ratio decreased by 6% during the 50 000-generation experiment. Because the evolved and ancestral bacteria were grown under identical conditions, our measurements reflect only evolved, heritable changes. Previous work has shown that *E. coli* cells also exhibit a plastic response to variation in nutrient supply, with their C : P ratio decreasing approximately 25% in response to a reduced C : P supply ratio, while the C : N supply ratio was held constant and the C : N ratio in biomass did not change [44]. The time scale of our experiment, while long in comparison to other laboratory experiments, is extremely brief in the context of Earth's history. Overall, our results indicate that evolutionary changes in stoichiometry can occur over a period of years or decades, and these evolved changes can be of similar scope to short-term physiological responses.

As predicted given the carbon-limited medium, we observed significant declines in both the C : N and C : P ratios of the bacterial biomass. However, there was no evidence of direct selection for elemental sparing, because the proportion of carbon (which was the limiting element) in biomass did not change. Rather, the declines in the C : N and C : P ratios resulted from increases in the proportions of both nitrogen and phosphorus in the bacteria. These increases might reflect a relaxation of prior selection for elemental sparing of nitrogen and phosphorus. However, we cannot distinguish the direct effect of selection due to low carbon, high nitrogen and high phosphorus from the indirect effects of selection favouring other traits in the evolution experiment. For example, some portion of the stoichiometric changes that we observed might simply be correlated responses to selection for larger cell size [45], faster growth rate [31] or other traits.

The exceptionally high C : N and C : P ratios of the evolved clone from the citrate-consuming lineage, which had access to approximately 10 times more carbon than any other population [34], provide some evidence that the declines in the C : N and C : P ratios in the other populations were beneficial specifically under the very low C : N and C : P supply ratios of the LTEE. However, the higher relative carbon content of the citrate-consuming clone is not necessarily itself strictly an evolutionary response. Instead, the citrate consumer's higher carbon content might also be, in whole or in part, a plastic physiological response to the higher carbon availability that it experiences as a result of its evolved ability to consume citrate.

Given the carbon-limited conditions of the LTEE, one might reasonably expect that the strongest selection on the bacteria would be to reduce the carbon in their biomass. However, increases in the bacteria's nitrogen and phosphorus content drove the changes in the C : N and C : P ratios, while there was no significant change in the percentage of carbon in the biomass. Taken at face value, this finding suggests that the proportion of carbon in biomass may be less evolutionarily flexible than the proportions of nitrogen and phosphorus. Alternatively, similar absolute changes in carbon content may be more difficult to detect because carbon makes up a much larger portion of the biomass.

Although the proportion of carbon in biomass did not change appreciably, the absolute amount of carbon that accumulated in the biomass increased significantly in the evolved bacteria (figure 2*b*). This increase in carbon was associated with an increase in total biomass (figure 2*c*). The increase in carbon retention was not caused by an increase in the amount of carbon consumed, because all of the bacteria (with the exception of the citrate consumer that was excluded from this analysis) consumed the same 25 mg l^−1^ of glucose. Therefore, to retain more carbon in biomass, the bacteria must have released less carbon, either as carbon dioxide or as by-products excreted into the medium (and not then recycled for growth). Our results parallel those of an experiment in which yeast evolved under carbon limitation for 350 generations [21]. The evolved yeast retained more carbon in their biomass, but the proportion of carbon in the biomass was unchanged. It may be that carbon-use efficiency (the proportion of carbon consumed that is incorporated into biomass) is more evolutionarily flexible than the proportion of carbon relative to other elements in biomass, at least in some organisms and contexts.

In this regard, it is also interesting that the LTEE provides no evidence of a trade-off between growth rate and carbon-use efficiency. Instead, the evolved bacteria have increased in both their growth rate [31,46] and carbon-use efficiency (this study; see also [31,46,47] for indirect evidence based on optical density and total biovolume of cells, rather than on biomass). A trade-off between growth rate and yield is a common pattern in microbial life-history traits [48–51] (but see [52]). Under the LTEE's well-mixed conditions, selection for increased growth rate is strong and direct, but there is no direct selection for increased yield [31]. For example, a mutant that grows faster, despite using resources less efficiently, would have a competitive advantage in the LTEE because the reduced resource availability would affect all competitors equally. Thus, it is surprising to observe an increase in biomass. It appears, therefore, that the ancestor was sufficiently maladapted to the conditions of the LTEE that both growth rate and yield could increase [46].

The temporal trends in stoichiometry in population Ara-1 over 60 000 generations were broadly consistent with the LTEE populations as a whole (figure 3). (Because the LTEE had reached 60 000 generations when we measured the temporal trajectories for this population, the data extend beyond the 50 000 generations analysed across the full set of populations.) The greater resolution of the Ara-1 dataset allowed us to observe whether the rate of stoichiometric change varied over time. Stoichiometry changed more rapidly during the early generations and more slowly later on. There was no indication, however, that the direction of the stoichiometric trends changed over time. The shapes of the stoichiometric trajectories roughly match those observed for fitness and cell size over time in the LTEE [28,53], suggesting that fitness, cell size and stoichiometry are closely linked in the LTEE. Recent work has shown that the fitness of populations in the LTEE is still increasing [28,54]. Similarly, the C : N ratio continued to decline between 5000 and 60 000 generations, indicating that stoichiometry can evolve over long periods of time. While the changes in C : P and N : P during that time frame were not statistically significant, the directional trends were consistent with earlier declines.

One possible explanation for the increased nitrogen and phosphorus content of the evolved bacteria would be higher levels of nucleic acids, which are rich in nitrogen and phosphorus compared to other cellular components [5]. We expected to see higher nucleic acid content in the evolved cells, even during stationary phase, because they exhibit a shorter lag phase and faster exponential growth following the daily transfers into fresh medium [31]. Higher ribosomal copy number and therefore also nucleic acid content are often associated with rapidly growing cells. A higher nucleic acid content during stationary phase might also allow for a faster transition to growth. However, we did not observe any significant change in the nucleic acid content. The proportion of cellular phosphorus we measured in nucleic acids was much lower than reported in previous experiments with *E. coli* [44], probably because our measurements were made during stationary phase rather than exponential growth. Both DNA and RNA levels are closely tied to the growth rate in bacteria, because the number of genome copies and the transcription rate are higher in rapidly growing cells [55,56]. Therefore, our results do not provide a test of the growth-rate hypothesis, which focuses on growing organisms. In any case, the low nucleic acid content, together with the lack of significant change in the nucleic acid content per biomass, indicates that the overall increase in the percentage of phosphorus in evolved cells reflects changes in cellular components other than nucleic acids.

Given the constancy of the nucleic acid content per biomass at stationary phase, the observed changes in stoichiometry presumably result from changes in the abundance of proteins and other broad classes of cellular components that differ in their elemental content. Higher nitrogen and phosphorus content might reflect increased storage of those elements in molecules such as amino acids, glutamine and polyphosphate, which would allow faster growth after the daily transfer into fresh medium. Indeed, reduced levels of polyphosphate have been shown to increase the duration of the lag phase of *E. coli* [57]. Over longer time scales than the LTEE has run thus far, selection for elemental sparing might even change the composition of individual proteins or other specific components. We calculate that a mutation that shifts a protein residue from a carbon-rich amino acid to a carbon-poor amino acid would take several hundred years to approach fixation in the LTEE (see the electronic supplementary material). Such mutations are unlikely to have contributed to the changes in stoichiometry observed so far, but this calculation indicates that selection on stoichiometry could continue over much longer time periods.

Our results show the potential for experimental evolution studies to shed new light on changes in stoichiometry. Our work shows that stoichiometry can evolve over a few thousand generations in response to a new environment, and that it continues to evolve for many tens of thousands of generations. These results raise the possibility that large-scale changes in elemental availability in Earth's ecosystems, for example, caused by climate change or increased fertilizer use, could drive evolutionary changes in organismal stoichiometry. Shifts in organismal stoichiometry could, in turn, alter the effects of anthropogenic change on ecological interactions and ecosystem processes. Stoichiometric evolution might be usefully incorporated into eco-evolutionary models, such as those that forecast the response of global phytoplankton communities to climate change [7,58] or nutrient cycling by heterotrophic bacteria [59–61]. In order to model and predict the evolution of stoichiometry, however, we will need to know much more about how stoichiometry responds to selection and what environmental factors generate selection on stoichiometry. Experimental evolution offers a valuable tool for acquiring that knowledge.

## Supplementary Material

Calculation of selection and fixation time for carbon-sparing amino-acid changes

## Data accessibility

Data were deposited in the Dryad Digital Repository http://dx.doi.org/10.5061/dryad.m53r1 [62].

## References

[1] Vanni MJ, Flecker AS, Hood JM, Headworth JL 2002 Stoichiometry of nutrient recycling by vertebrates in a tropical stream: linking species identity and ecosystem processes. Ecol. Lett. 5, 285–293. (doi:10.1046/j.1461-0248.2002.00314.x)

[2] Klausmeier CA, Litchman E, Daufresne T, Levin SA 2004 Optimal nitrogen-to-phosphorus stoichiometry of phytoplankton. Nature 429, 171–174. (doi:10.1038/nature02454) 15141209 10.1038/nature02454

[3] Bertram S, Bowen M, Kyle M, Schade J 2008 Extensive natural intraspecific variation in stoichiometric (C: N: P) composition in two terrestrial insect species. J. Insect Sci. 8, 26 (doi:10.1673/031.008.2601) 10.1673/031.008.2601 PMC3061598 20298114

[4] Zimmerman AE, Allison SD, Martiny AC 2014 Phylogenetic constraints on elemental stoichiometry and resource allocation in heterotrophic marine bacteria. Environ. Microbiol. 16, 1398–1410. (doi:10.1111/1462-2920.12329) 24237481 10.1111/1462-2920.12329

[5] Sterner RW, Elser JJ 2002 Ecological stoichiometry: the biology of elements from molecules to the biosphere. Princeton, NJ: Princeton University Press.

[6] Elser J 2006 Biological stoichiometry: a chemical bridge between ecosystem ecology and evolutionary biology. Am. Nat. 168, S25–S35. (doi:10.1086/509048) 17109326 10.1086/509048

[7] Munday PL, Warner RR, Monro K, Pandolfi JM, Marshall DJ 2013 Predicting evolutionary responses to climate change in the sea. Ecol. Lett. 16, 1488–1500. (doi:10.1111/ele.12185) 24119205 10.1111/ele.12185

[8] Gorokhova E, Dowling TE, Weider LJ, Crease TJ, Elser JJ 2002 Functional and ecological significance of rDNA intergenic spacer variation in a clonal organism under divergent selection for production rate. Proc. R. Soc. Lond. B 269, 2373–2379. (doi:10.1098/rspb.2002.2145) 10.1098/rspb.2002.2145 PMC1691159 12495506

[9] Schlüter L, Lohbeck KT, Gutowska MA, Gröger JP, Riebesell U, Reusch TB 2014 Adaptation of a globally important coccolithophore to ocean warming and acidification. Nat. Clim. Change 4, 1024–1030. (doi:10.1038/nclimate2379)

[10] Velmurugan N, Sung M, Yim SS, Park MS, Yang JW, Jeong KJ 2014 Systematically programmed adaptive evolution reveals potential role of carbon and nitrogen pathways during lipid accumulation in *Chlamydomonas reinhardtii*. Biotechnol. Biofuels 7, 117 (doi:10.1186/s13068-014-0117-7) 25258645 10.1186/s13068-014-0117-7 PMC4174265

[11] Kay AD, Ashton IW, Gorokhova E, Kerkhoff AJ, Liess A, Litchman E 2005 Toward a stoichiometric framework for evolutionary biology. Oikos 109, 6–17. (doi:10.1111/j.0030-1299.2005.14048.x)

[12] Merchant SS, Helmann JD 2012 Elemental economy: microbial strategies for optimizing growth in the face of nutrient limitation. Adv. Microb. Physiol. 60, 91 (doi:10.1016/b978-0-12-398264-3.00002-4) 22633059 10.1016/B978-0-12-398264-3.00002-4 PMC4100946

[13] Bragg JG, Wagner A 2007 Protein carbon content evolves in response to carbon availability and may influence the fate of duplicated genes. Proc. R. Soc. B 274, 1063–1070. (doi:10.1098/rspb.2006.0290) 10.1098/rspb.2006.0290 PMC2124476 17264057

[14] Quigg A, Finkel ZV, Irwin AJ, Rosenthal Y, Ho T-Y, Reinfelder JR, Schofield O, Morel FM, Falkowski PG 2003 The evolutionary inheritance of elemental stoichiometry in marine phytoplankton. Nature 425, 291–294. (doi:10.1098/rspb.2010.1356) 13679916 10.1038/nature01953

[15] Reinhard CT et al. 2017 Evolution of the global phosphorus cycle. Nature 541, 386–389. (doi:10.1038/nature20772) 28002400 10.1038/nature20772

[16] Acquisti C, Elser JJ, Kumar S 2009 Ecological nitrogen limitation shapes the DNA composition of plant genomes. Mol. Biol. Evol. 26, 953–956. (doi:10.1093/molbev/msp038) 19255140 10.1093/molbev/msp038 PMC2727375

[17] Elser JJ 2006 Signatures of ecological resource availability in the animal and plant proteomes. Mol. Biol. Evol. 23, 1946–1951. (doi:10.1093/molbev/msl068) 16870683 10.1093/molbev/msl068

[18] Günther T, Lampei C, Schmid KJ 2013 Mutational bias and gene conversion affect the intraspecific nitrogen stoichiometry of the *Arabidopsis thaliana* transcriptome. Mol. Biol. Evol. 30, 561–568. (doi:10.1093/molbev/mss249) 23115321 10.1093/molbev/mss249

[19] Baudouin-Cornu P, Surdin-Kerjan Y, Marliere P, Thomas D 2001 Molecular evolution of protein atomic composition. Science 293, 297–300. (doi:10.1126/science.1061052) 11452124 10.1126/science.1061052

[20] Gounand I et al. 2016 Size evolution in microorganisms masks trade-offs predicted by the growth rate hypothesis. Proc. R. Soc. B 283, 20162272 (doi:10.1098/rspb.2016.2272) 10.1098/rspb.2016.2272 PMC5204171 28003453

[21] Goddard MR, Bradford MA 2003 The adaptive response of a natural microbial population to carbon- and nitrogen-limitation. Ecol. Lett. 6, 594–598. (doi:10.1046/j.1461-0248.2003.00478.x)

[22] Frisch D, Morton PK, Chowdhury PR, Culver BW, Colbourne JK, Weider LJ, Jeyasingh PD 2014 A millennial-scale chronicle of evolutionary responses to cultural eutrophication in *Daphnia*. Ecol. Lett. 17, 360–368. (doi:10.1111/ele.12237) 24400978 10.1111/ele.12237

[23] Roy Chowdhury P, Frisch D, Becker D, Lopez JA, Weider LJ, Colbourne JK, Jeyasingh PD 2015 Differential transcriptomic responses of ancient and modern *Daphnia* genotypes to phosphorus supply. Mol. Ecol. 24, 123–135. (doi:10.1111/mec.13009) 25410011 10.1111/mec.13009

[24] Declerck SA, Malo AR, Diehl S, Waasdorp D, Lemmen KD, Proios K, Papakostas S 2015 Rapid adaptation of herbivore consumers to nutrient limitation: eco-evolutionary feedbacks to population demography and resource control. Ecol. Lett. 18, 553–562. (doi:10.1111/ele.12436) 25913306 10.1111/ele.12436

[25] Schaum C-E, Rost B, Collins S 2016 Environmental stability affects phenotypic evolution in a globally distributed marine picoplankton. ISME 10, 75–84. (doi:10.1038/ismej.2015.102) 10.1038/ismej.2015.102 PMC4681848 26125683

[26] Godwin CM, Cotner JB 2014 Carbon: phosphorus homeostasis of aquatic bacterial assemblages is mediated by shifts in assemblage composition. Aquat. Microb. Ecol. 73, 245–258. (doi:10.3354/ame01719)

[27] Godwin CM, Cotner JB 2015 Aquatic heterotrophic bacteria have highly flexible phosphorus content and biomass stoichiometry. ISME 9, 2324–2327. (doi:10.1038/ismej.2015.34) 10.1038/ismej.2015.34 PMC4579474 25798755

[28] Wiser MJ, Ribeck N, Lenski RE 2013 Long-term dynamics of adaptation in asexual populations. Science 342, 1364–1367. (doi:10.1126/science.1243357) 24231808 10.1126/science.1243357

[29] Lenski RE, Rose MR, Simpson SC, Tadler SC 1991 Long-term experimental evolution in *Escherichia coli*. I. Adaptation and divergence during 2,000 generations. Am. Nat. 138, 1315–1341. (doi:10.1086/285289)

[30] Tenaillon O et al. 2016 Tempo and mode of genome evolution in a 50,000-generation experiment. Nature 536, 165–170. (doi:10.1038/nature18959) 27479321 10.1038/nature18959 PMC4988878

[31] Vasi F, Travisano M, Lenski RE 1994 Long-term experimental evolution in *Escherichia coli*. II. Changes in life-history traits during adaptation to a seasonal environment. Am. Nat. 144, 432–456. (doi:10.1086/285685)

[32] Jeong H et al. 2009 Genome sequences of *Escherichia coli* B strains REL606 and BL21 (DE3). J. Mol. Biol. 394, 644–652. (doi.org/10.1016/j.jmb.2009.09.052) 19786035 10.1016/j.jmb.2009.09.052

[33] Davis BD, Mingioli ES 1950 Mutants of *Escherichia coli* requiring methionine or vitamin B12. J. Bacteriol. 60, 17–28. 15436457 10.1128/jb.60.1.17-28.1950 PMC385836

[34] Blount ZD, Borland CZ, Lenski RE 2008 Historical contingency and the evolution of a key innovation in an experimental population of *Escherichia coli*. Proc. Natl Acad. Sci. USA 105, 7899–7906. (doi:10.1073/pnas.0803151105) 18524956 10.1073/pnas.0803151105 PMC2430337

[35] Blount ZD, Barrick JE, Davidson CJ, Lenski RE 2012 Genomic analysis of a key innovation in an experimental *Escherichia coli* population. Nature 489, 513–518. (doi:10.1038/nature11514) 22992527 10.1038/nature11514 PMC3461117

[36] Bertani G 1951 Studies on lysogenesis. I. The mode of phage liberation by lysogenic *Escherichia coli*. J. Bacteriol. 62, 293–300. 14888646 10.1128/jb.62.3.293-300.1951 PMC386127

[37] Aspila K, Agemian H, Chau A 1976 A semi-automated method for the determination of inorganic, organic and total phosphate in sediments. Analyst 101, 187–197. (doi:10.1039/an9760100187) 1259177 10.1039/an9760100187

[38] US Environmental Protection Agency. 1997 Methods for the determination of chemical substances in marine and estuarine environmental samples method 440.0. Washington, DC: US Environmental Protection Agency.

[39] Maddamsetti R, Lenski RE, Barrick JE 2015 Adaptation, clonal interference, and frequency-dependent interactions in a long-term evolution experiment with *Escherichia coli*. Genetics 200, 619–631. (doi:10.1534/genetics.115.176677) 25911659 10.1534/genetics.115.176677 PMC4492384

[40] Wielgoss S et al. 2013 Mutation rate dynamics in a bacterial population reflect tension between adaptation and genetic load. Proc. Natl Acad. Sci. USA 110, 222–227. (doi:10.1073/pnas.1219574110) 23248287 10.1073/pnas.1219574110 PMC3538217

[41] Khan AI, Dinh DM, Schneider D, Lenski RE, Cooper TF 2011 Negative epistasis between beneficial mutations in an evolving bacterial population. Science 332, 1193–1196. (doi:10.1126/science.1203801) 21636772 10.1126/science.1203801

[42] de Visser JAG, Lenski RE 2002 Long-term experimental evolution in *Escherichia coli*. XI. Rejection of non-transitive interactions as cause of declining rate of adaptation. BMC Evol. Biol. 2, 19 (doi:10.1186/1471-2148-2-19) 12443537 10.1186/1471-2148-2-19 PMC134600

[43] Barrick JE, Yu DS, Yoon SH, Jeong H, Oh TK, Schneider D, Lenski RE, Kim JF 2009 Genome evolution and adaptation in a long-term experiment with *Escherichia coli*. Nature 461, 1243–1247. (doi:10.1038/nature08480) 19838166 10.1038/nature08480

[44] Makino W, Cotner J, Sterner R, Elser J 2003 Are bacteria more like plants or animals? Growth rate and resource dependence of bacterial C:N:P stoichiometry. Funct. Ecol. 17, 121–130. (doi:10.1046/j.1365-2435.2003.00712.x)

[45] Mongold JA, Lenski RE 1996 Experimental rejection of a nonadaptive explanation for increased cell size in *Escherichia coli*. J. Bacteriol. 178, 5333–5334. (doi:10.1128/jb.178.17.5333-5334.1996) 8752359 10.1128/jb.178.17.5333-5334.1996 PMC178338

[46] Novak M, Pfeiffer T, Lenski RE, Sauer U, Bonhoeffer S 2006 Experimental tests for an evolutionary trade-off between growth rate and yield in *E. coli*. Am. Nat. 168, 242–251. (doi:10.1086/506527) 16874633 10.1086/506527

[47] Lenski RE, Mongold JA 2000 Cell size, shape, and fitness in evolving populations of bacteria. In Scaling in biology (eds Brown JH, West GB), pp. 221–235. Oxford, UK: Oxford University Press.

[48] Bachmann H, Fischlechner M, Rabbers I, Barfa N, Branco dos Santos F, Molenaar D, Teusink B 2013 Availability of public goods shapes the evolution of competing metabolic strategies. Proc. Natl Acad. Sci. USA 110, 14 302–14 307. (doi:10.1073/pnas.1308523110) 10.1073/pnas.1308523110 PMC3761572 23940318

[49] Pfeiffer T, Schuster S, Bonhoeffer S 2001 Cooperation and competition in the evolution of ATP-producing pathways. Science 292, 504–507. (doi:10.1126/science.1058079) 11283355 10.1126/science.1058079

[50] MacLean R 2008 The tragedy of the commons in microbial populations: insights from theoretical, comparative and experimental studies. Heredity 100, 471–477. (doi:10.1038/sj.hdy.6801073) 18449959

[51] Meyer JR, Gudelj I, Beardmore R 2015 Biophysical mechanisms that maintain biodiversity through trade-offs. Nat. Commun. 6, 6278 (doi:10.1038/ncomms7278) 25695944 10.1038/ncomms7278

[52] Reding-Roman C, Hewlett M, Duxbury S, Gori F, Gudelj I, Beardmore R 2017 The unconstrained evolution of fast and efficient antibiotic-resistant bacterial genomes. Nat. Ecol. Evol. 1, 0050 (doi:10.1038/s41559-016-0050) 10.1038/s41559-016-0050 28812723

[53] Lenski RE, Travisano M 1994 Dynamics of adaptation and diversification: a 10,000-generation experiment with bacterial populations. Proc. Natl Acad. Sci. USA 91, 6808–6814. (doi:10.1073/pnas.91.15.6808) 8041701 10.1073/pnas.91.15.6808 PMC44287

[54] Lenski RE et al. 2015 Sustained fitness gains and variability in fitness trajectories in the long-term evolution experiment with *Escherichia coli*. Proc. R. Soc. B 282, 20152292 (doi:10.1098/rspb.2015.2292) 10.1098/rspb.2015.2292 PMC4707762 26674951

[55] Akerlund T, Nordström K, Bernander R 1995 Analysis of cell size and DNA content in exponentially growing and stationary-phase batch cultures of *Escherichia coli*. J. Bacteriol. 177, 6791–6797. (doi:10.1128/jb.177.23.6791-6797.1995) 7592469 10.1128/jb.177.23.6791-6797.1995 PMC177544

[56] Schaechter M, Maaløe O, Kjeldgaard N 1958 Dependency on medium and temperature of cell size and chemical composition during balanced growth of *Salmonella typhimurium*. J. Gen. Microbiol. 19, 592–606. (doi:10.1099/00221287-19-3-592) 13611202 10.1099/00221287-19-3-592

[57] Crooke E, Akiyama M, Rao NN, Kornberg A 1994 Genetically altered levels of inorganic polyphosphate in *Escherichia coli*. J. Biol. Chem. 269, 6290–6295. 8119977

[58] Follows MJ, Dutkiewicz S, Grant S, Chisholm SW 2007 Emergent biogeography of microbial communities in a model ocean. Science 315, 1843–1846. (doi:10.1126/science.1138544) 17395828 10.1126/science.1138544

[59] Franklin O, Hall EK, Kaiser C, Battin TJ, Richter A 2011 Optimization of biomass composition explains microbial growth-stoichiometry relationships. Am. Nat. 177, E29–E42. (doi:10.1086/657684) 21460549 10.1086/657684

[60] Sinsabaugh RL, Follstad Shah JJ 2012 Ecoenzymatic stoichiometry and ecological theory. Annu. Rev. Ecol. Evol. Syst. 43, 313–343. (doi:10.1146/annurev-ecolsys-071112-124414)

[61] Allison S 2012 A trait-based approach for modelling microbial litter decomposition. Ecol. Lett. 15, 1058–1070. (doi:10.1111/j.1461-0248.2012.01807.x) 22642621 10.1111/j.1461-0248.2012.01807.x

[62] Turner CB, Wade BD, Meyer JR, Sommerfeld BA, Lenski RE 2017 Data from: Evolution of organismal stoichiometry in a long-term experiment with *Escherichia coli*. Dryad Digital Repository. (doi:10.5061/dryad.m53r1) 10.1098/rsos.170497 PMC5541568 28791173

