# Supplementary material for: Evolution of organismal stoichiometry in a long-term experiment with Escherichia coli
Source: R Soc Open Sci. 2017 Jul 19;4(7):170497. doi: 10.1098/rsos.170497 (PMC5541568; doi:10.1098/rsos.170497)
Supplement: Calculation of selection and fixation time for carbon-sparing amino-acid changes [file rsos170497supp1.pdf]

Supplementary information for: Evolution of organismal stoichiometry in a long-term experiment with *Escherichia coli*

Authors: Caroline B. Turner, Brian D. Wade, Justin R. Meyer, Brooke A. Sommerfeld, and Richard E. Lenski

Journal: *Royal Society Open Science*

Calculation of selection and fixation time for carbon-sparing amino-acid changes

We can calculate the effect of individual mutations on the elemental content of proteins, and thereby the expected selective benefit of an amino-acid substitution under the carbon-sparing hypothesis. Similar logic should apply for other cellular components. Comparative studies have suggested that selection can change the amino-acid content of proteins, reducing an organism's use of the most limiting elements [1, 2]. However, the fitness benefit of any mutation that changes a single amino acid is likely to be extremely small. Following the approach of Bragg and Wagner [3], and given the effective population size (N_e) for the LTEE of 3.3 x 10^7 [4], we calculate that mutations that save a single carbon atom would be visible to selection, such that $s > 1/(2N_e)$ [5], in proteins with more than ~450 copies per cell. Individual amino-acid changes, specifically tryptophan to glycine, can save as many as 9 carbon atoms per protein molecule [3]. A mutation that saved 9 carbon atoms per protein molecule could be selected for in proteins with ~50 or more copies per cell. Of course, such a mutation would require a very long time to achieve fixation. As an example, consider a mutation that saves 9 carbon atoms per molecule in an abundant protein with 10,000 copies per cell. Even after escaping drift and without competition from other beneficial mutations, this mutation would require on the order of 10^6 generations (~400 years) to approach fixation in the population.

References:

1. Baudouin-Cornu, P., Surdin-Kerjan, Y., Marliere, P., Thomas, D. 2001 Molecular evolution of protein atomic composition. *Science*. **293**, 297-300. (10.1126/science.1061052)
2. Elser, J. 2006 Biological stoichiometry: A chemical bridge between ecosystem ecology and evolutionary biology. *American Naturalist*. **168**, S25-S35.
3. Bragg, J. G., Wagner, A. 2009 Protein material costs: single atoms can make an evolutionary difference. *Trends in Genetics*. **25**, 5-8. (10.1016/j.tig.2008.10.007)
4. Lenski, R. E., Rose, M. R., Simpson, S. C., Tadler, S. C. 1991 Long-term experimental evolution in *Escherichia coli*. I. Adaptation and divergence during 2,000 generations. *Am. Nat.* **138**, 1315-1341. (10.1086/285289)
5. Kimura, M. 1983 *The neutral theory of molecular evolution*. Cambridge, UK: Cambridge University Press.
